# Water‐Derived Radical Species Initiate Six‐Electron Oxidation of Cyclic Ketones in Microdroplets

**DOI:** 10.1002/anie.1888676

**Published:** 2026-07-16

**Authors:** Muhammad Mujahid Ali, Manish Jana, William LeFever, Robert Graham Cooks

**Affiliations:** ^1^ Department of Chemistry Purdue University West Lafayette Indiana USA

**Keywords:** accelerated reactions, mass spectrometry, microdroplets, oxidation, sustainable chemistry

## Abstract

Direct six‐electron oxidation of cyclohexanone and other cyclic ketones and associated C‐C bond cleavage can be achieved in microdroplets without the need for external oxidants, metal catalysts, or thermal activation. Mechanistic studies indicate that presence of trace amounts of water initiate the oxidative transformation by generating reactive radical species. Isotopic labeling experiments employing D_2_O and H_2_^18^O provide evidence for involvement of water radical cation (H_2_O^•+^) as the active oxidant. Experiments using different nebulizing gases suggest that both water radical cation and molecular oxygen are required for the oxidation. Furthermore, the radical trapping agent 2,2,6,6‐tetramethylpiperidone (TEMPO) suppresses product formation, consistent with a radical‐mediated mechanism. The substrate scope of this reaction was verified using different cyclic ketones which yielded dicarboxylic acids/keto acids. In line with the mass spectrometric results, the reaction using cyclohexanone was scaled up using a single sprayer (∼ 12 mg product was collected in 3 h) for characterization by ^1^H NMR spectroscopy, which verified the formation of adipic acid in microdroplets. Overall, this study (i) demonstrates the need for trace amounts of water during microdroplet reactions, (ii) showcases the exceptional oxidizing power of water microdroplets, and (iii) introduces a green, energy‐efficient pathway for dicarboxylic acid/keto acid synthesis.

## Graphical abstract

Spontaneous six‐electron oxidation of cyclic ketones to yield dicarboxylic acids/keto acids is achieved in ethanol microdroplets containing about 2% water under ambient conditions without any metal salts, acids, or oxidants. Mechanistic studies show that trace amounts of water and molecular oxygen serve as active oxidants in this transformation.

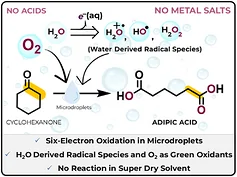

## 1 Introduction

Microdroplet chemistry has emerged as a powerful platform for accelerating chemical reactions [1, 2, 3, 4, 5], unveiling reactivity patterns that are occasionally distinct from those in bulk solutions. When bulk liquids are dispersed into micro‐ or nanometer‐sized droplets, the combination of a large surface‐to‐volume ratio and strong interfacial electric fields generates a distinctive reaction environment [6, 7, 8, 9]. The remarkable reactivity of microdroplets arises from several interrelated properties, (a) partial solvation of reactants [10, 11, 12, 13], (b) strong surface electric fields [14, 15, 16], and (c) ordered molecular orientation [17, 18]. Redox reactions are prominent in microdroplets, and oxidation is a frequently observed reaction type because the unique combination of interfacial effects, charge accumulation, and radical generation make oxidative processes thermodynamically and kinetically favorable [19]. The strong fields ionize water, creating a super acidic or super basic outmost surface layer, depending on field direction. Field‐induced ionization of water and other organic solvents under the strong interfacial electric field [20], together with the unique interfacial environment, promotes the formation of reactive oxygen species (ROS). Several oxidizing agents have been proposed in aqueous microdroplet based redox chemistry, including water radical cation (H_2_O^•+^) [21, 22, 23, 24, 25, 26, 27], hydroxyl radical (^•^OH) [28, 29, 30, 31], and hydrogen peroxide (H_2_O_2_) [32, 33]. Among these species, the water radical cation is an ultra‐strong oxidant with an estimated redox potential of greater than 3 V versus the Nernst Hydrogen Electrode (NHE) [34]. It is thought to be stabilized at the relatively dry (low solvation) microdroplet surface, while it rapidly decays to give H_3_O^+^ and ^•^OH in bulk water [35, 36]. The secondary ROS, hydroxyl radical (^•^OH), can generate hydrogen peroxide (H_2_O_2_) [10, 20]. The powerful oxidizing capability of microdroplets has been used previously to efficiently convert organic molecules like alcohols [37], aldehydes [38], and ketones [39] to their corresponding acids, and thiols to their corresponding disulfides and sulfinates [40, 41, 42, 43].

**SCHEME 1:**
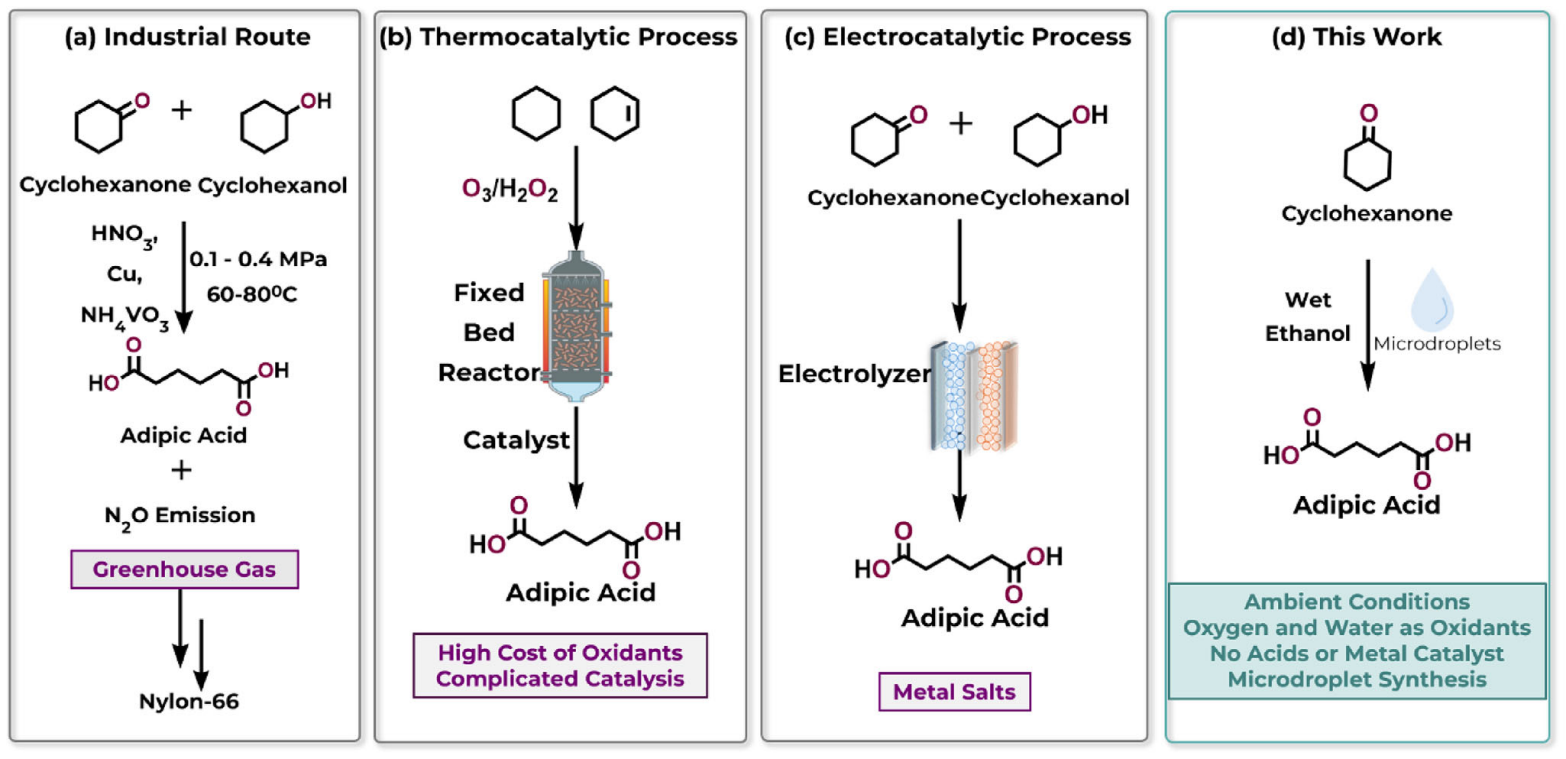
Adipic acid synthesis by different approaches: (a) industrial process using KA oil [47, 48], (b) thermocatalytic oxidation of cyclohexane and cyclohexene [50, 52], (c) electrochemical oxidation of KA oil [44, 53, 54], and (d) microdroplet‐based method developed in this work.

Dicarboxylic acids are vital platform chemicals used to make polymers like nylon and polyesters, and serve as building blocks for pharmaceuticals, food additives (flavor enhancers), and fragrances. Here we focus on one dicarboxylic acid, that is, adipic acid. It is a key monomer conventionally synthesized by catalytic oxidation of ketone‐alcohol (KA) oil at elevated temperatures and is used in the production of nylon‐66, plasticizers, and polyurethanes [44, 45]. The global demand for adipic acid continues to rise, with a projected market value of nearly USD 6 billion by 2030 [46]. Currently, more than 90% of adipic acid is produced by thermal oxidation of ketone–alcohol oil (KA oil), a mixture of cyclohexanol and cyclohexanone (Scheme 1a) [47, 48]. In this process, cyclohexanol is first oxidized to cyclohexanone using a copper‐ammonium metavanadate catalyst, followed by nitric acid oxidation (50%–65%) to yield adipic acid. However, this route generates large amounts of nitrous oxide (N_2_O), a potent greenhouse gas [49]. Greener alternatives utilizing hydrogen peroxide (H_2_O_2_) have been explored (Scheme 1b) [50], but their effectiveness is limited by the instability of H_2_O_2_ under elevated temperature and pH [51, 52]. More recently, the electrocatalytic oxidation of KA oil to adipic acid using water as the oxygen source and renewable electricity as the energy input (Scheme 1c) has gained attention as a greener alternative [53, 54, 55]. However, these processes still rely on metal salts or electrodes to facilitate electron transfer, increasing cost, adding potential metal waste, and reducing their overall sustainability.

Here, we report the six‐electron oxidation of cyclohexanone and other cyclic ketones in electrosprayed microdroplets to produce dicarboxylic acids/keto acids under ambient conditions. These reactions take advantage of the oxidative power of the charged droplet interface, allowing efficient conversion of cyclic ketones to dicarboxylic acids. Mechanistic studies suggest that water‐derived radical species are the primary oxidants in driving the oxidation, with atmospheric oxygen contributing to one step in the process. The substrate scope for this reaction was verified using seven other cyclic ketones that gave dicarboxylic acids/keto acids upon oxidation. The reaction of cyclohexanone was scaled up to collect products for ^1^H NMR analysis, which confirmed the successful formation of adipic acid. This reaction proceeded in a scaled‐up version at a rate of 4 mg/h using a single sprayer.

## 2 Results and Discussion

### 2.1 Adipic Acid Formation in Microdroplets

**FIGURE 1:**
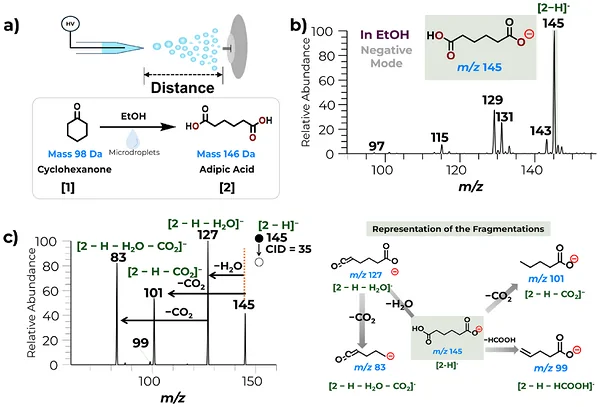
(a) Schematic of the microdroplet based oxidation of cyclohexanone (**1**) to adipic acid (**2**) in microdroplets. (b) Negative‐ion mass spectrum with intermediates generated during the reaction. (c) MS/MS spectrum of *m/z* 145 (**[2 − H**]**^−^**) and suggested structures of fragment ions.

A 10 mM solution of cyclohexanone (**1,** mass 98 Da) in EtOH was sprayed from a nano electrospray ionization (nESI) capillary using a voltage of −2 kV to generate charged droplets. Figure 1a presents an overview of the microdroplet‐based strategy and the reaction studied in this work. Immediately upon sample introduction, the mass spectrum exhibited a peak at *m/z* 145 corresponding to the formation of deprotonated adipic acid [**2 − H**]^−^ (Figure 1b). Signals at *m/z* 115, 129, and 131 were also observed, and are assigned (below) to partially oxidized intermediates formed during stepwise oxidation and ring‐opening of cyclohexanone. The low abundance of the precursor ion at *m/z* 97 (negative ion mode) is attributed to the poor ionization efficiency of cyclohexanone.

To validate the product ion structure as well as to confirm the absence of any weakly bonded species, tandem mass spectrometry (MS/MS) was performed for *m/z* 145 ([**2 − H**]^−^); Figure 1c. The MS/MS spectrum of product ion at *m/z* 145 at a relatively high energy (collision‐induced dissociation, CID 30 units) showed loss of water (m/z 127) ([**2 − H − H_2_O**]**^−^**) and carbon dioxide (*m*/*z* 101, [**2 − H − CO_2_**]^−^). Peaks at *m/z* 83 and m/z 99 were also observed in the MS/MS spectra. The structures of the fragment ions are rationalized in Figure 1c. The MS/MS spectrum for *m/z* 145 ion matches that of standard adipic acid [56].

**FIGURE 2:**
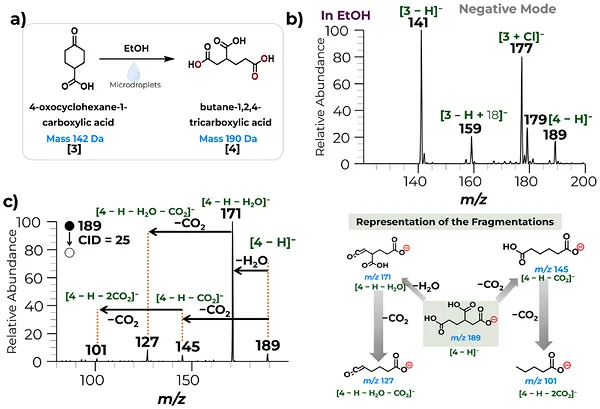
(a) Microdroplet based oxidation of 4‐oxocyclohexanecarboxylic acid (**3**) to butane‐1,2,4‐tricarboxylic acid (**4**). (b) Mass spectrum of 10 mM of 4‐oxocyclohexanecarboxylic acid (**3**) recorded in negative ion mode shows formation of product at *m/z* 189 ([**4 − H**]^−^). (c) MS/MS spectrum of *m/z* 189 and suggested structures of fragment ions.

As the ionization efficiency of cyclohexanone (**1**) in the negative ion mode is too low for reliable quantitation, 4‐oxo‐cyclohexanecarboxylic acid (**3**, mass 142 Da) was selected as a model precursor to obtain approximate quantitative information on product formation using the MS‐based conversion ratio [12]. As illustrated in Figure 2, electrospraying a solution of 4‐oxocyclohexanecarboxylic acid (**3**) in ethanol similarly results in generation of peaks at *m/z* 141, corresponding to [**3 − H**]^−^, *m/z* 189 (product, **[4 − H**]**^−^**) as well as the chlorinated adducts of **3** ([**3 + Cl**]^−^, *m/z* 177, 179).

Structural confirmation was obtained through MS/MS of the *m/z* 189 ion ([**4 − H**]^−^, Figure 2c). Fragmentation of this ion produced diagnostic neutral losses of water and carbon dioxide to generate peaks at *m/z* 171 ([**4 − H − H_2_O**]^−^) and *m/z* 145 ([**4 − H − CO_2_**]^−^). The other peaks in the MS/MS spectrum at *m/z* 127 and *m/z* 101 are due to subsequent losses of CO_2_ from *m/z* 171 and 145, respectively. The fragmentation pattern is consistent with characteristics of carboxylic acids [55]. These results demonstrate that 4‐oxocyclohexanecarboxylic acid (**3**) undergoes rapid oxidative ring‐opening in microdroplets to yield a tricarboxylic acid derivative, 3‐carboxy‐adipic acid (**4**), within milliseconds of droplet flight.

### 2.2 Solvent and Distance Effects on the Oxidation Reaction

The effect of solvent during the oxidation of 4‐oxocyclohexanecarboxylic acid (**3**) was examined using six different solvents: water, methanol, ethanol, isopropanol, acetonitrile (ACN), and acetone. As shown in Figure S1, aqueous ethanol gave the highest intensity of product ion (*m/z* 189), indicating an optimal balance between solubility, volatility, conductivity, and reactivity. Pure water as the solvent showed no detectable product formation (*m/z* 189 was absent), likely due to the limited solubility of the hydrophobic organic reagent and mass‐transport limitations in the aqueous phase. In contrast, experiments with the addition of a trace amount of water to ethanol maintain high solubility of the organic reactants while retaining water molecules within the interfacial region of the microdroplets. The resulting mixed‐solvent interface may facilitate reactant encounters with activated water species and so promote the observed reaction. Methanol and isopropanol gave moderate conversions. Acetone exhibited minimal product formation in the mass spectrometric experiment due to its low polarity and the poor stability of the spray. In the case of ACN as solvent, the product ion intensity was very low, and it gave several side products due to possible side reactions of ACN in the presence of water and cyclohexanone (Figure S2) [57]. Overall, these findings indicate that the oxidation of cyclohexanone occurs at the air‐solution interface of microdroplets (a fact also supported by the lack of the corresponding bulk reaction in ethanol). They also suggest that solvent polarity and volatility significantly influence these microdroplet‐based oxidation reactions by modulating interfacial charge distribution, droplet size, and lifetime.

In the case of microdroplet reactions, the relative product ion intensity is expected to increase with increasing distance between the sprayer tip and the inlet of the mass spectrometer [58, 59]. Hence, we analyzed the effect of distance on the oxidation of 4‐oxocyclohexanecarboxylic acid (**3**) using EtOH + 2% H_2_O as spray solvent to understand how droplet flight time and coulombic fission (decreased droplet size) influence product formation. At short inlet to sprayer distances (5–13 mm), only the reactant ion (*m/z* 141) was observed in the mass spectrum, indicating little to no reaction (Figure S3). However, as the spray distance was increased, the relative intensity of the product ion (*m/z* 189) increased, in relative (but not absolute) terms indicating more oxidation. In many other reactions, smaller droplets with high surface to volume ratios also are responsible for greater product formation. The highest conversion (ratio of products to precursor, product, and intermediates) was seen at a sprayer distance of 135 mm from the mass spectrometer inlet. At a distance beyond 135 mm, the absolute intensity of the product ion at *m/z* 189 is too low to allow measurement of the conversion ratio.

From the mass spectrum, we found only 3% conversion of 4‐oxocyclohexanecarboxylic acid (**3**) to the butane‐1,2,4‐tricarboxylic acid (**4**) product at a sprayer to inlet distance of 5 mm. However, as the distance between the sprayer tip and inlet increased, the conversion ratio increased to 32%. A discussion of conversion ratio determination [12, 59] and how the conversion ratio changes with distances can be found in the supporting information, Figure S3. However, this relative method is not a quantitative measure of product formation. In the case of cyclohexanone (**1**), since the ionization efficiency is low, the conversion ratio was determined using an internal standard having a more comparable ionization efficiency to that of adipic acid. This is discussed below.

### 2.3 Quantitative Measurement of the Adipic Acid Products

For quantitation of adipic acid produced by the oxidation of cyclohexanone, decanoic acid (**5**) was selected as an internal standard due to the absence of spectral overlap in the negative ion mode. Experiments revealed that decanoic acid exhibited measurably lower ionization efficiency than adipic acid at the same concentration. To compensate for this difference, calibration solutions were prepared at a 1:10 molar ratio of adipic acid to decanoic acid (0.5 and 5 mM, respectively), which produced comparable ion intensities for both species. The concentrations of adipic acid were varied from 5 to 0.025 mM while maintaining a constant 5 mM concentration of decanoic acid (spectra are shown in Figure S4). The calibration curve displayed excellent linearity (*R* ^2^ = 0.99) with equation *y* = 2.034*x* + 0.014. To correct for cluster ion formation, the intensity of monomer ions was used directly, while dimer ion intensities were multiplied by two. Based on this calibration, the concentration of adipic acid generated from cyclohexanone (5 mM) oxidation was determined at a sprayer to inlet distance of 35 mm.

### 2.4 Mechanistic Investigation

**FIGURE 3:**
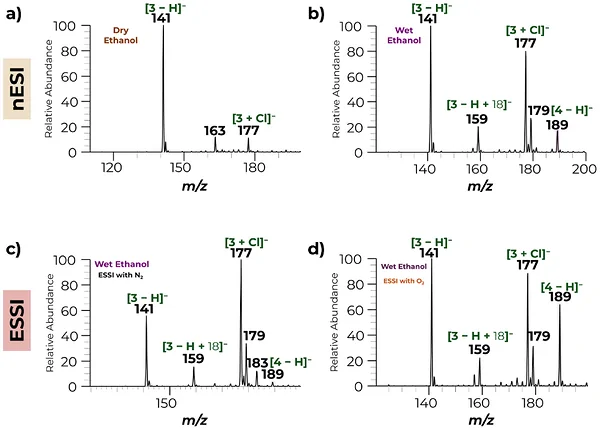
Mass spectrum of 4‐carboxy‐cyclohexanone (**3**) recorded using nESI in (a) super dry ethanol and (b) in wet ethanol (ethanol containing 2% water). Mass spectrum of 4‐carboxy‐cyclohexanone (**3**) recorded using ESSI with (c) nitrogen and (d) oxygen as nebulizing gases.

**FIGURE 4:**
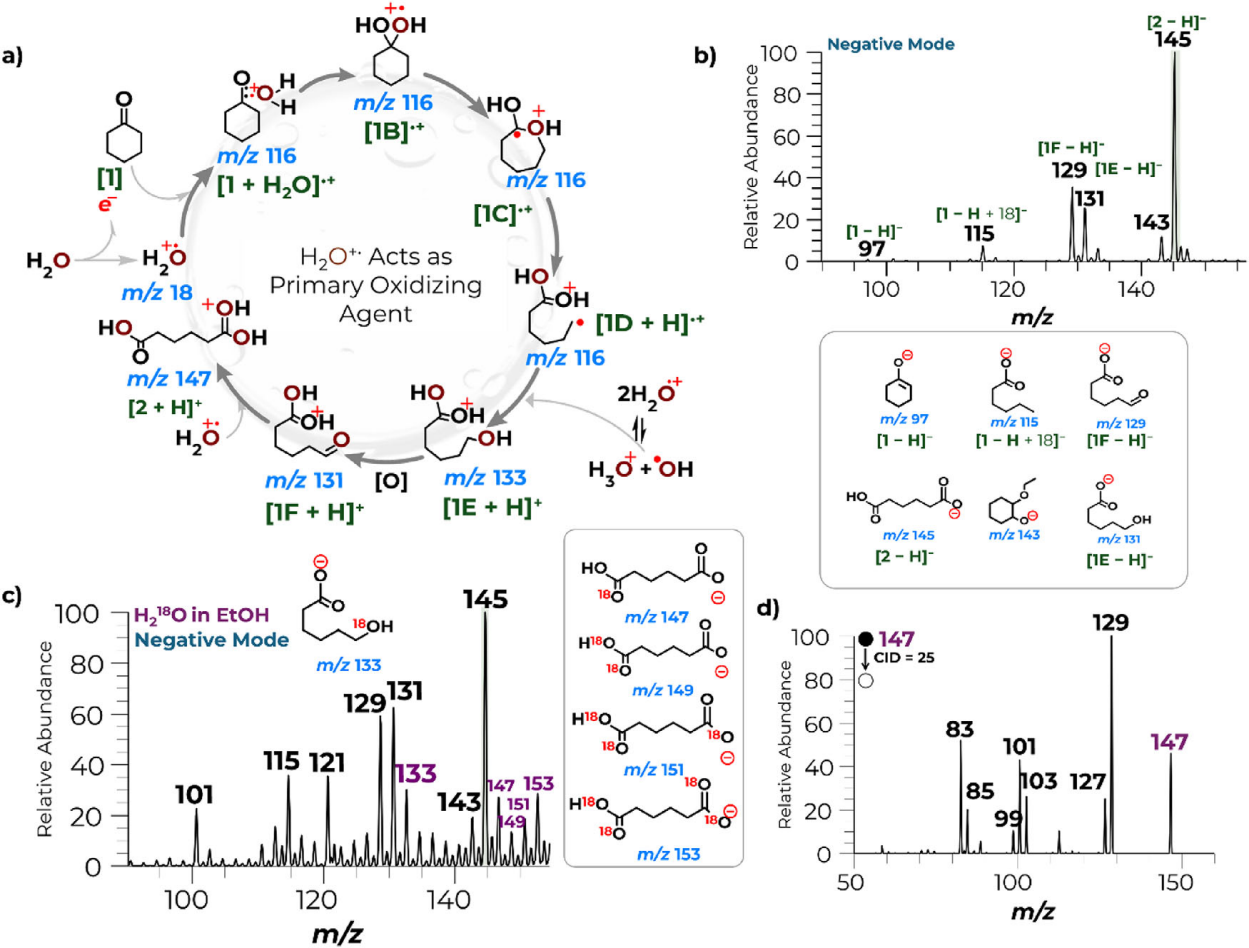
(a) Proposed mechanism for the oxidation of cyclohexanone to adipic acid involving water radical cation as the oxidizing agent. (b) Mass spectrum and corresponding intermediates during the oxidation of cyclohexanone (negative ion mode). (c) Addition of H_2_^18^O in dry ethanol during the oxidation shows incorporation of ^18^O in the reaction intermediates as well as the final product in the negative ion mode. (d) Negative ion mode MS/MS spectrum for *m/z* 147 (the single ^18^O incorporated product).

- Role of water: It has been noted in the literature that a certain amount of water is essential to generate the super‐acidic/basic conditions necessary for microdroplet reactions [59, 60]. To investigate if water was required for this reaction, we dried ethanol thoroughly over 3Å molecular sieves for one week under nitrogen atmosphere to eliminate all water. This included purging the anhydrous ethanol solution containing molecular sieves gently with nitrogen gas for an hour followed by keeping it under a nitrogen atmosphere for a week. Next, we nano‐electrosprayed 4‐carboxy‐cyclohexanone (**3**) dissolved in super‐dry ethanol (special care must be taken during preparation of the solution and filling the capillary, as even brief exposure of the solvent to the atmosphere can lead to moisture absorption) [61]. Under these conditions, the mass spectrum in the negative ion mode showed no product (*m/z* 189) and no peak corresponding to the water adduct at *m/z* 159 ([**3** – H + 18]^−^) (Figure 3a) (note H_2_^18^O data below). When 2% water was added to the dry ethanol, and the solution was nano‐electrosprayed, the peaks corresponding to *m/z* 159 ([**3** – H + 18]^−^) and the product ion (*m/z* 189) were observed in the mass spectrum (Figure 3b). This observation was further confirmed using dry ACN as the solvent and N_2_ as the nebulizing gas in electrosonic spray ionization (ESSI), where no product formation was detected (Figure S2a). Only a trace amount of product was observed upon introducing 2% water into the dry ACN under nitrogen atmosphere, as shown in Figure S2b.
- Role of nebulizing gases: Next, we investigated the role of nebulizing gases during this reaction. We conducted the reaction by dissolving 4‐carboxy‐cyclohexanone (**3**) in wet (2% water) ethanol and electrosprayed it (ESSI) using nitrogen and oxygen separately as the nebulizing gases. When nitrogen was used as a nebulizing gas, the product ion at *m/z* 189 appeared with very low intensity (Figure 3c). In contrast, when oxygen was used as the nebulizing gas, the intensity of the *m/z* 189 peak increased significantly (Figure 3d). These findings indicate that both water and oxygen are necessary for the efficient oxidation of cyclohexanone during microdroplet reactions.
- Role of hydrogen peroxide: Several studies by Zare et al. have proposed that hydrogen peroxide (H_2_O_2_) acts as the active oxidant in microdroplet‐based oxidation reactions [62, 63, 64]. To test this, H_2_O_2_ was added with 4‐carboxy cyclohexanone (**3**) in ethanol (10 mM each) and sprayed directly into the mass spectrometer. The addition of hydrogen peroxide did not enhance product formation (Figure S5). This result is consistent with a recent report where the addition of hydrogen peroxide to KA oil did not generate adipic acid or other oxidized products in microdroplets [65].
- Involvement of radical species in the reaction mechanism: To investigate the formation of reactive radical species during the oxidation reaction, we mixed varying equivalents of the radical scavenger 2,2,6,6‐tetramethylpiperidone (TEMPO) to 4‐carboxy‐cyclohexanone and directly analyzed the mixture using mass spectrometry as depicted in Figure S6. The addition of TEMPO led to a significant decrease in the intensity of the intermediate oxidation peak at *m/z* 159 ([**3** – H + 18]^−^) due to capture of the radical reagent species, while the fully oxidized product (ion at *m/z* 189) was completely absent. The total suppression of product formation in the presence of a radical quencher provides strong, albeit indirect, experimental evidence that a radical pathway is indeed the primary mechanistic route.
- Proposed mechanism: Molecular ionization by water radical cations for oxidation in atmospheric aerosols, and small‐scale synthesis has been noted previously [66]. Based on previous reports and experimental findings of this work, we propose a mechanism for the oxidation of cyclohexanone (**1**) to adipic acid (**2**) in microdroplets involving both water radical cation and oxygen as the active oxidants during the process (Figure 4a). Initially, the water radical cation oxidizes the ketone to generate a ketyl radical cation intermediate [**1** + H_2_O]^•+^. In the next step, hydroxyl radical cation undergoes a migratory ring insertion, forming a cyclic hemiacetal‐like structure [**1C**]^•+^. Subsequent C–O bond cleavage yields a terminal radical cation [**1D** + H]^•+^, which captures a hydroxyl radical (^•^OH) formed via disproportionation of the water radical cation dimer (2H_2_O^• +^ H_3_O^+^+^•^OH), producing 6‐hydroxyhexanoic acid [**1E** + H]^+^. In the next step, molecular oxygen (O_2_) oxidizes the hydroxyl group to a corresponding aldehyde, forming 6‐oxohexanoic acid [**1F** + H]^+^ (we note that this assignment is tentative, since experiments using ^18^O_2_ as the nebulizing gas were not performed). Finally, water radical cation (H_2_O^•+^) oxidizes the aldehyde to the corresponding carboxylic acid, yielding adipic acid as the final product [**2** + H]^+^. Note that the involvement of hydroxy radical (^•^OH) in this reaction is not excluded. Water radical cation (H_2_O^•+^) acts as the primary oxidant in this reaction, which can subsequently generate hydroxyl radicals (^•^OH) as well as hydrogen peroxide (H_2_O_2_).

Although the mechanism as presented here (Figure 4a) is in the positive ion mode, the mass spectra of the reactive intermediates as well as the product were recorded in negative ion mode since the carboxylic acids can be detected readily in the negative ion mode [67]. The mass spectrum of cyclohexanone (**1**) to adipic acid (**2**) and corresponding structures of the intermediates are shown in Figure 4b. The apparent six‐electron oxidation in this case occurs through a series of controlled single‐electron transfers, with each radical contributing one electron at a time. No individual radical accounts for the entire oxidation sequence.

f. Isotope labeling study using H_2_^18^O and D_2_O: To probe the mechanism further, we used H_2_^18^O as well as D_2_O during our experiments. Although ethanol is a polar protic solvent capable of proton exchange with labeled water, the reaction gave less conversion to product in ACN (Figure S2); therefore, ethanol was selected as the solvent for the isotope labeling experiments. Involvement of water during the reaction was confirmed by adding D_2_O in ethanolic solution of cyclohexanone (detected in the positive ion mode) (Figure S7). Peaks at *m/z* 117, 118 in positive ion mode were observed when D_2_O was used with EtOH (Figure S7). Similarly, when 2% H_2_^18^O was added to ethanolic solution of cyclohexanone (Figure 4c), new peaks appeared in the mass spectrum at *m/z* 133, 147, 149, 151 and 153 in negative ion mode, and at *m/z* 118 in positive ion mode. MS/MS on these newly observed ions confirmed the incorporation of ^18^O into the final product (Figures 4d and S8–S11).

To further validate the involvement of water in the six‐electron oxidation mechanism, a standard solution of adipic acid in ethanol was prepared. 2% H_2_^18^O and D_2_O were separately added to the ethanolic solution and sprayed into the mass spectrometer. In these cases, no incorporation of ^18^O and very little incorporation of deuterium (D) was observed in the final adipic acid product (Figure S12) [68]. This confirms that water participates in the reaction mechanism and acts as an active oxidant in the oxidation of cyclohexanone (**1**) to adipic acid (**2**). Isotope labeling occurs specifically during the reaction, rather than after product formation. Additionally, the involvement of oxygen during this reaction is supported by the increased product yield observed when oxygen is used as the nebulizing gas instead of nitrogen (Figure 3d). A proposed mechanism based on involvement of hydroxyl radical (^•^OH) is provided in the supporting information (Figure S13).

### 2.5 Substrate Scope

**FIGURE 5:**
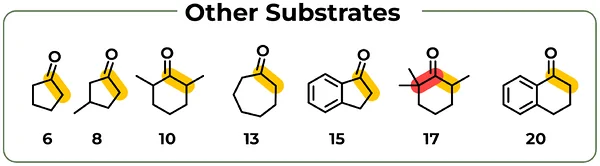
Cyclic ketones used for oxidation reaction.

The application of this oxidation reaction has been extended to seven other cyclic ketones (substituted and unsubstituted, Figure 5). The mass spectrum and the MS/MS of these compounds were provided in the supporting information Figures S14–S20. The unsubstituted ketones produced dicarboxylic acids, while the substitution at the α‐ position yielded keto acids. The presence of electron donating substituents on the α‐ position of the cyclic ketones stabilize the radical species (**[1D + H]^+^**, shown in Figure 4a) to a greater extent and lead to greater conversion of the cyclic ketones to the corresponding keto acids (Figures S16 and S19). In contrast, the reaction did not proceed in presence of electron withdrawing substituent on the α‐ position, verified using 2‐chloro cyclohexanone, where the corresponding dicarboxylic acid/keto acid formation in microdroplet was not observed. These results support the suggested mechanism but limit the application of this microdroplet based oxidation method.

#### 2.5.1 Scale up for Characterization by ^1^H NMR

**FIGURE 6:**
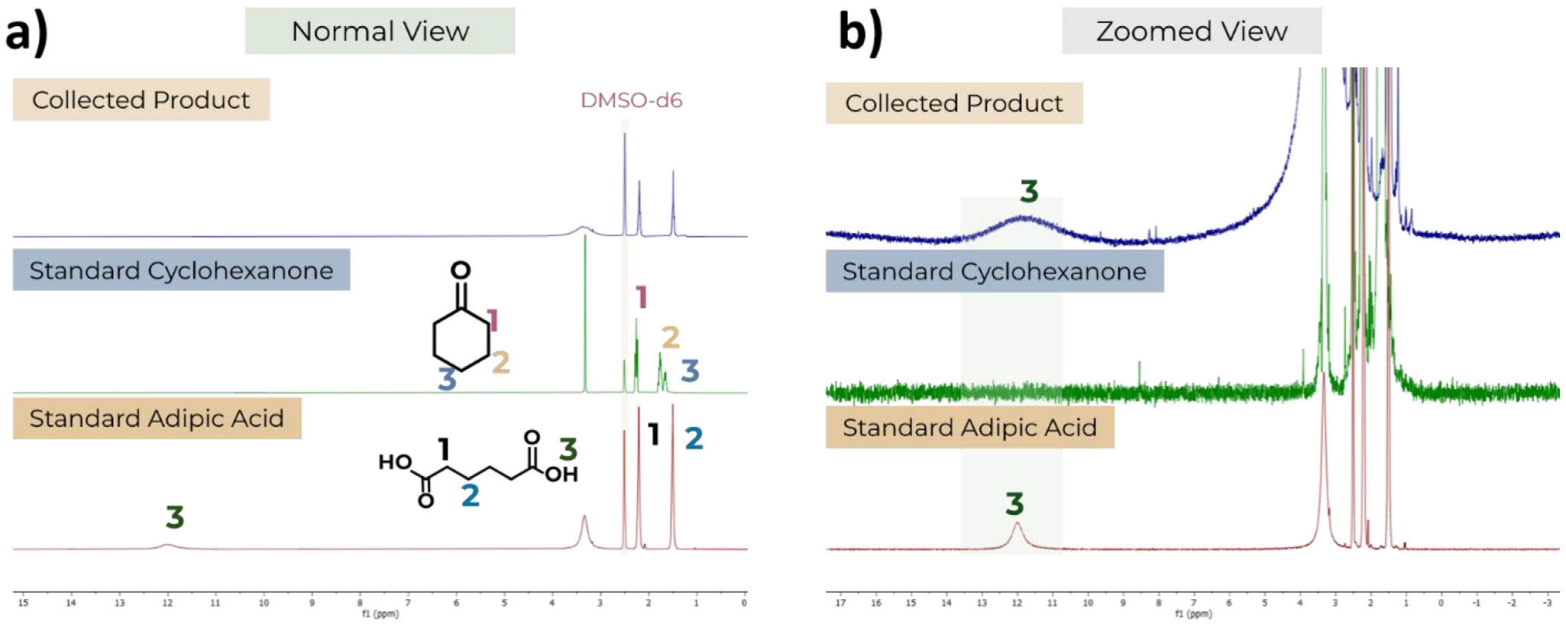
Stacked ^1^H NMR spectra of standard adipic acid (orange), cyclohexanone (green) and the collected product after spraying (blue) (a) normal view and (b) zoomed view showing the presence of a broad peak at ∼ *δ* 12 ppm for the carboxylic acid proton.

To test whether the observed transformation occurs as a discrete microdroplet‐phase phenomenon and is not an artifact of the mass spectrometry ionization process, we performed the experiment off‐line and used NMR for characterization of collected reaction products. For characterization by ^1^H NMR, the oxidation of cyclohexanone (**1**) to adipic acid (**2**) was selected and scaled up using an electrosonic sprayer at 50 µL min^−1^ operated under a negative potential of −3 kV and collected in a Pyrex bottle. This bottle contained an electrically grounded surface to provide favorable deposition conditions. The distance between the sprayer tip and the grounded surface was kept at ∼15 cm. The details of the scale up experiment were given in supporting information. A 100 mM cyclohexanone solution (9 mL, 87 mg) was continuously sprayed at a flow rate of 50 µL min^−1^ for 3 h and approximately 12 mg of product was collected. The collected product was redissolved in DMSO‐d_6_ and analyzed by ^1^H NMR without any further purification. The corresponding ^1^H NMR spectrum of the collected product is shown in Figure 6 as well as in Figure S21. The higher vapor pressure of cyclohexanone relative to adipic acid facilitates clean conversion, yielding product (adipic acid) without residual starting materials or significant amounts of by‐products [69]. This represents a clear advantage of microdroplet method over previously reported methods, which often rely on harsher reaction conditions and require additional purification of the collected products [46, 47, 49, 52, 53].

For comparison, ^1^H NMR spectra of standard adipic acid (Figure S22) and cyclohexanone (Figure S23) were also recorded in DMSO‐d_6_. Notably, the characteristic proton signal in ^1^H NMR at *δ* 12.0 ppm for carboxylic acid was absent in pure cyclohexanone but appeared prominently in both the standard adipic acid as well as in the sprayed and collected product, confirming the generation of adipic acid in microdroplets (Figure 5b, zoomed view). We also stirred a solution of cyclohexanone in EtOH for 24 h in the presence of oxygen. Then solvent was evaporated and the residue was dissolved in DMSO‐d_6_ for NMR analysis. The NMR spectra (Figure S24) did not show any sign of product formation. These results provide further evidence of adipic acid formation under ambient conditions via oxidation of cyclohexanone in wet ethanol microdroplets. We note that the rate of product formation should be significantly improved by using multiplexed sprayers as well as solvent recycling systems [70].

It is well established that microdroplets generated by electrospray or other nebulization process are inherently non‐equilibrium systems [14, 15, 16]. As these droplets rapidly evaporate, the surface charge density increases continuously (approaching the Rayleigh limit), which produces a high interfacial electric field (ranging from > 10^7^ to 10^9^ V/m). This time‐dependent field prevents the system from reaching a static, “bulk‐like” equilibrium state during the droplet's milliseconds lifespan. The observed six‐electron oxidation and the associated radical formation require an energy input much higher than *k*_B_ *T* at room temperature. In the absence of external thermal or photo‐excitation, the ultra‐high electric field (> 10^7 ^V/m) at the microdroplet interface, as supported by recent studies, remains the only physically plausible source of energy for these transformations [14, 15, 16, 71, 72, 73]. Partial solvation of reactants at the air‐water interface provides a “chemical driver” that, when coupled with reagents produced by the electric field, overcomes the thermodynamic barrier observed in bulk solution. The microdroplet interface functions not merely as a locus for reagents and solvent, but as a high‐energy reactor that facilitates complex multi‐electron transfers by fundamentally altering the transition‐state energetics.

While the sub‐nanosecond dynamics of electron tunnelling at the liquid‐air interface cannot be visualized directly with current spectroscopic methods, the total suppression of product formation in the presence of a radical quencher (Figure S6) strongly suggests that radical formation is the primary mechanistic pathway. Isotope labeling experiments further support the involvement of water radical cation species in the reaction, although we do not exclude the possibility that hydroxyl radicals might also act as active oxidants in the microdroplets (Figure S13). We also note that there is considerable current debate on the role of the water radical cation [22, 23, 24, 25, 26, 27, 34, 66]. However, we continue to hypothesize that water radical cation is the key radical species in this chemistry under electrospray conditions.

We also assert that the observed transformation is a discrete microdroplet‐based phenomenon, not an artifact of the mass spectrometry ionization process, as a referee has asked. By microdroplet phenomenon we consider charged microdroplets in their ordinary state which includes Taylor cone formation and coulombic fission and note that mass spectrometers have no essential role. Evidence for this is the following:

1. We have successfully performed off‐line NMR spectroscopic characterization of the collected reaction products (Figure 6). NMR requires milligram‐scale quantities—orders of magnitude greater than what is produced during transient gas‐phase ion‐molecule reactions. The successful collection and structural analysis of the product confirms that the chemistry occurs within the liquid microdroplets during nebulization and the droplet flight time, independent of any mass spectrometer related vacuum environment.
2. The Taylor cone interface is highly polarized [74]. To verify that radical formation and the subsequent reactions occur at the droplet interface rather than involving electrochemical effects at the emitter electrode interface, we conducted the reaction using ESSI with oxygen as the nebulizing gas, without applying external voltage during droplet generation. The mass spectrum shown in Figure S25 demonstrates product formation without an applied voltage, confirming that the reaction occurs at the droplet interface. However, since the Taylor cone can form transiently—during Coulombic explosions [75]—we accept the possibility of radical species being formed at the Taylor cone interface.

## 3 Conclusion

This study demonstrates a sustainable, metal‐free, and ambient‐conditions route for the six‐electron oxidation of cyclic ketones to dicarboxylic acids/keto acids within electrospray‐generated microdroplets. The reaction is primarily driven by the great oxidizing power of the water derived radical species at the droplet interface, with atmospheric oxygen also making an essential contribution to one step in the overall oxidation process. The lack of product formation in dry solvents underscores the essential role of trace water in microdroplet chemistry. Mass spectrometric analysis along with isotopic labelling experiments using D_2_O and H_2_^18^O confirmed the role of water in formation of dicarboxylic acid along with key intermediates, while ^1^H NMR characterization provided further support for product formation as a discrete microdroplet‐phase phenomenon not as an artifact of the mass spectrometry ionization process. The observed product formation in the absence of an applied voltage supports the conclusion that the reaction occurs at the droplet interface, rather than through electrochemical reaction at the electrode interface. Although the overall yield is relatively low due to limited reaction time in the droplets, the process highlights the remarkable oxidative capability and selectivity achievable under ambient microdroplet conditions. Compared to conventional high‐temperature nitric acid oxidation, this approach eliminates the need for harsh oxidants and significantly reduces environmental impact. It also eliminates the need for purification steps after product collection. Overall, these findings showcase the remarkable oxidative capacity of microdroplets as promising green oxidizing agents for developing sustainable oxidation processes and synthesizing industrially important compounds.

## Author Contributions

**Muhammad Mujahid Ali**: conceptualization, methodology, formal analysis, data curation, writing – review and editing. **Manish Jana**: conceptualization, methodology, formal analysis, data curation, writing – review and editing, writing – original draft. **William Lefever**: writing – review and editing, formal analysis. **Robert Graham Cooks**: conceptualization, funding acquisition, writing – review and editing, visualization, supervision, resources.

## Conflicts of Interest

The authors declare no conflicts of interest.

## Supporting information




**Supporting File 1**: anie73674‐sup‐0001‐SuppMat.docx.

## Data Availability

The raw data that support the findings of this study are available from the corresponding author upon reasonable request.
