# Supplementary material for: Water‐Derived Radical Species Initiate Six‐Electron Oxidation of Cyclic Ketones in Microdroplets
Source: Angew Chem Int Ed Engl. 2026 Jul 16;65(39):e1888676. doi: 10.1002/anie.1888676 (PMC13592315; doi:10.1002/anie.1888676)
Supplement: Supplementary file 1 [file ANIE-65-e1888676-s001.docx]

**Supporting Information**

**Water-derived Radical Species Initiate Six-Electron Oxidation of Cyclic Ketones in Microdroplets**

Muhammad Mujahid Ali, ^†[a]^ Manish Jana, ^†[a]^ William LeFever, ^[a]^ Prof. R. Graham Cooks*^[a]^

^[a]^ Department of Chemistry, Purdue University, 560 Oval Drive, West Lafayette, Indiana-47907

^†^ These authors have contributed equally to this work.

Email: [cooks@purdue.edu](mailto:cooks@purdue.edu)

**General Information**

Cyclohexanone (**1**), adipic acid (**2**), 4-oxocyclohexanecarboxylic acid (**3**), decanoic acid (**5**), cyclopentanone (**6**), 3-methylcyclopentanone (**8**), 2,6-dimethylcyclohexanone (**10**), cycloheptanone (**13**), indanone (**15**), 2,2,6-trimethylcyclohexanone (**17**), α-tetralone (**20**) were all purchased from Sigma-Aldrich (St. Louis, MO, USA). HPLC-grade water was obtained from Fisher Scientific (Hampton, NH, USA). Methanol (MeOH, HPLC grade, ≤0.1% water), isopropanol (IPA, HPLC grade, ≤0.2% water), and acetonitrile (ACN, LC/MS grade, ≤0.1% water) were also purchased from Fisher Scientific. Anhydrous ethanol was obtained from Decon Laboratories, Inc. (King of Prussia, PA, USA). Molecular sieves (3 Å, beads) were purchased from Sigma-Aldrich. They were activated at 200°C prior to use. For experiments with extra dry solvent, anhydrous ethanol (≥99.5%, 200 proof) was obtained from Sigma-Aldrich. This ethanol was transferred under a nitrogen atmosphere into an oven-dried, septum-sealed 20 mL vial containing pre-activated 3Å molecular sieves. The solution was gently purged with nitrogen for one hour and then stored under positive nitrogen pressure for one week. Special care was also taken during preparation of the sample solutions involving anhydrous ethanol.

High-purity nitrogen (N₂) and oxygen (O₂) gases (≥99.999%) were obtained from Indiana Oxygen Company (Indianapolis, IN, USA) and used for nebulization during the experiments as well as in the scale up experiment. Isotopically labeled water (H₂¹⁸O, ¹⁸O 10%) and deuterium oxide (D₂O, D, 99.9%) were purchased from Cambridge Isotope Laboratories (Tewksbury, MA, USA). (2,2,6,6-Tetramethylpiperidin-1-yl)oxyl (TEMPO, ≥98%) was obtained from Sigma-Aldrich.

All solvents and reagents were used as received without further purification. Borosilicate glass capillaries used for nanoelectrospray ionization mass spectrometry (nESI-MS) analysis (1.5 mm O.D., 0.86 mm I.D., 10 cm length) were obtained from Sutter Instruments (Novato, CA, USA). All analyses were performed using nESI coupled to a Finnigan LTQ linear ion trap mass spectrometer (Thermo Fisher Scientific, San Jose, CA, USA).

Nanoelectrospray emitters with an internal diameter of 5 μm were fabricated using a P-97 Micropipette Puller (Sutter Instruments, Novato, CA) and fitted into a stainless-steel electrode. A spray voltage of −2 kV was applied, with the distance between the emitter tip and MS inlet being varied from 3 to 135 mm depending on the experiment. We note that the magnitude of the applied potential (−2 kV) was not selected for any specific mechanistic reason. It was simply used to generate droplets. Because the products were dicarboxylic acids, they were readily detected in the negative ion mode. For solvent optimization, spectra were acquired at fixed distances of 5 mm and 135 mm using various solvents, including methanol, ethanol, isopropanol, acetone, acetonitrile and water. For quantitative measurements, the spray distance was fixed at 35 mm for all experiments. The MS inlet temperature was maintained at 250 °C, with a capillary voltage of 15 V and a tube lens voltage of 65 V. Tandem mass spectrometry (MS/MS) was employed for structural characterization of selected precursor ions.

The electrosonic spray ionization (ESSI) emitter was constructed using fused silica lines (150 µm I.D. and 363 µm O.D., purchased from Molex PolyMicro), one tee assembly, one union assembly, two Nano Tight sleeves, and a silica capillary (IDEX Health and Science). To maintain the flow of reagent solution at a rate of 5 µL/min, a syringe pump (Standard Infusion PHD 22/2000, Harvard Apparatus) was used with gastight syringe (Hamilton Robotics). For experiments in which products were directly analyzed by mass spectrometry N_2_ and O_2_ gases were used separately as the nebulizing gases at 40 psi.

¹H NMR spectra were recorded on a Bruker NEO300 spectrometer equipped with a 5 mm BBFO Z-gradient probe.

**Scale up Experiment**

A 200 mM solution of cyclohexanone was prepared and loaded into a glass syringe for the oxidation experiment at scale. The reaction was carried out using a 150 μm inner diameter fused silica capillary (Molex Polymicro; i.d. = 150 μm, o.d. = 353 μm) connected to a Harvard Apparatus PHD 2000 syringe pump operated in the infuse mode. The solution was continuously sprayed at a flow rate of 50 μL min⁻¹, while oxygen gas at 60 psi was used for nebulization. A negative potential of −3 kV was applied to the syringe. The aerosolized droplets were collected in a Pyrex bottle for 3 hours. A grounded copper foil was placed inside the Pyrex bottle during the collection process to facilitate collection of products. The collected product was subsequently redissolved in DMSO-d₆ for ¹H NMR analysis. No further purifications or chromatographic separations were performed on the product.

**Conversion Ratio Determination**:

The conversion ratio during the microdroplet cyclohexanone (**1**) to adipic acid (**2**) experiment was determined using an internal standard as discussed in the main manuscript. For 4-oxocyclohexanecarboxylic acid (**3**) the following equations were used to determine the conversion ratio as shown in Figure S3.

Here, I_P_, I_R_, I_INT_ are the ion intensities of the products, reactants and intermediates (respectively) formed during the microdroplet reaction. The third equation is the most stringent approach to calculate the conversion ratio since it discounts the intensity of the intermediates. Using equation **3** we have observed 32% conversion of the 4-oxocyclohexanecarboxylic acid (**3**) to butane-1,2,4-tricarboxylic acid (**4**) (inlet to sprayer distance of 135mm) as shown in Figure S3.

**Supporting Figures**

**Figure S1.** Mass spectra showing the effect of solvent on the microdroplet oxidation reaction of 4-oxocyclohexanecarboxylic acid **(3)** at two different distances (5 mm and 135 mm). To record MS spectra, 10 mM solution of 4-oxocyclohexanecarboxylic acid **(3)** was sprayed using n-ESI and negative voltage of −2 kV was employed on a steel electrode to generate droplets. Note that all spectra are normalized and that the absolute intensities decrease with increasing distances. Also note the general trend (except for water) of increased product formation with distance.

**Figure S2**. Mass spectrum of 4-oxocyclohexanecarboxylic acid **(3)** using (**a**) dry acetonitrile as solvent, showing no product formation (*m/z* 189, absent) and (**b**) acetonitrile + 2% H_2_O as solvent. showing product at *m/z* 189 together with side-products. Spectra recorded by ESSI with N_2_ as nebulizing gas.

**Figure S3.** Effect of spray-to-inlet distance on the microdroplet oxidation of 4-oxocyclohexanecarboxylic acid (**3**) to 1,2,4-butanetricarboxylic acid **(4)**. Increased spray distances (shown in respective spectra) enhanced the product ion (*m/z* 189) intensity relative to the reactant (*m/z* 141), indicating higher conversion to the product. The conversion ratios were calculated using Eq. (1) – (3) involving the ion ratios of the reactant (I_R_), Intermediates (I_INT_) and the product (I_P_) (NL indicates normalization level in the spectrum).

**Figure S4.** Quantitation of adipic acid (**2**) production by oxidation of cyclohexanone (**1**), using decanoic acid (**5**) as internal standard. Mass spectrum of adipic acid (**2**) at different concentrations from 5 mM to 0.025 mM while maintaining a constant 5 mM concentration of decanoic acid (**5**). Calibration curve displaying linearity (R^2^ = 0.99) with equation *y* = 2.034*x* + 0.014. To correct for dimer formation, the intensity of monomer ions was used directly, while dimer ion intensities were multiplied by two. Based on this calibration curve, the concentration of adipic acid generated from cyclohexanone (5 mM) oxidation was determined to be approximately 0.028 mM (inlet to sprayer distance of 35 mm).

**Figure S5.** Effect of addition of H_2_O_2_ during the oxidation of 4-oxocyclohexanecarboxylic acid (**3**). **a)** Mass spectrum for the reaction without addition of H_2_O_2_. **b)** Mass spectrum after addition of 10 mM H_2_O_2_ during the oxidation reaction (NL indicates normalization level in the spectrum, a measure of total ion signal).

**Figure S6.** Influence of the radical scavenger TEMPO (2,2,6,6-tetramethylpiperidone) on the oxidation of 4-carboxycyclohexanone (**3**) in microdroplets. (a) Mass spectra without addition of TEMPO, (b) after addition of 1 equivalent of TEMPO and (c) 3 equivalents of TEMPO. The presence of TEMPO led to a marked decrease in the *m/z* 159 peak ([**3 − H** +18]⁻), while the final oxidation product at m/z 189 completely disappeared. d) Although the intensity of the peak generated at *m/z* 314 after reaction between TEMPO and one of the reaction intermediates is low, the MS/MS spectrum of *m/z* 314 showed neutral losses of water and carbon dioxide (NL indicates normalization level in the spectrum, a measure of total ion signal).

**Figure S7.** Effect of water during the experiment using cyclohexanone (**1**) was verified using addition of D_2_O in ethanol. (a) Mass spectrum recorded (in wet EtOH) in positive ion mode showing *m/z* 116 and (b) corresponding MS/MS showing neutral loss of 17 Da. (c) Upon addition of D_2_O, deuterium incorporation is shown by the peak at *m/z* 117 while (d) the MS/MS spectrum of *m/z* 117 showed neutral losses of OH (mass = 17 Da) and OD (mass = 18 Da).

**Figure S8.** The role of water during the reaction of cyclohexanone (**1**) was verified upon addition of H_2_O^18^ in ethanol. The MS/MS of *m/z* 118 in the positive ion mode shows loss of 17 (OH) to give *m/z* 101, and 19 (^18^OH) to give *m/z* 99. As expected, no loss of 18 is recorded.

**Figure S9.** Isotopic labeling experiment confirming the incorporation of O^18^ into the oxidation product of cyclohexanone (**1**) in microdroplets. (a) MS recorded in negative ion mode upon addition of 2% H_2_O^18^ in EtOH. (b) MS/MS of deprotonated adipic acid ion at *m/z* 145 ([**2 − H**]⁻). (c) MS/MS of product ion at *m/z* 147 ([**M – H + 2**]⁻), indicating single O¹⁸ incorporation into the final product. (d) MS/MS of product ion at *m/z* 149 ([**M – H + 4**]⁻), indicating double O¹⁸ incorporation in the final product. (e) MS/MS of product ion at *m/z* 151 ([**M – H + 6**]⁻), indicating triple O¹⁸ incorporation into the final product. (d) MS/MS of product ion at *m/z* 153 ([**M – H + 8**]⁻), indicating presence of all isotopically labelled O¹⁸ in the final product. (Note that MS/MS of *m/z* 151 ion shows a peak at *m/z* 136. This peak was also detected in blank ethanol (without any sample), shown in the next figure).

**Figure S10**: MS/MS spectrum for ion at *m/z* 151 in pure EtOH without any sample being present (background), showing a peak at *m/z* 136. This peak has also been observed in MS/MS of *m/z* 151 during isotope labelling experiment (**Figure S9, e**).

**Figure S11**: a) MS/MS spectrum for *m/z* 131 (cyclohexanone in EtOH). (b) MS/MS spectrum for *m/z* 133 (observed after addition of H_2_^18^O in EtOH during adipic acid formation in microdroplets).

**Figure S12. a**) Mass spectra (recorded in negative ion mode) of pure adipic acid dissolved in ethanol containing 2% H₂¹⁸O. No shift in the *m/z* 145 peak was observed, confirming that ¹⁸O was not incorporated into the molecule. **b)** Mass spectra (recorded in negative ion mode) of pure adipic acid dissolved in ethanol containing 2% D_2_O. MS showed little incorporation of D in the final product. These results indicate that once the product (adipic acid) is formed, isotopically labelled water is not incorporated under these experimental conditions.

**Figure S13.** Proposed mechanism for oxidation of cyclohexanone based on involvement of hydroxy radical(^•^OH).

**Figure S14.** a) Oxidation of cyclopentanone (**6**) to glutaric acid (**7**) in ethanol microdroplets. b) Mass spectrum recorded in negative ion mode showed formation of glutaric acid (**[7 − H]**^−^, *m/z* 131). c) MS/MS of *m/z* 117 and the suggested structures of fragment ions. d) MS/MS of *m/z* 131 and the suggested structures of fragment ions.

**Figure S15.** a) Oxidation of 3-methylcyclopentanone (**8**) to 3-methylpentanedioic acid (**9**) in ethanol microdroplets. b) Mass spectrum recorded in negative ion mode showing formation of 3-methylpentanedioic acid (**[9 − H]**^−^, *m/z* 145). c) MS/MS of *m/z* 131 and the suggested structures of fragment ions. d) MS/MS of *m/z* 145 and the suggested fragment ions structures.

**Figure S16.** a) Oxidation of 2,6-dimethylcyclohexanone (**10**) to give 2-methyl-6-oxoheptanoic acid (**11**) and 6-hydroxy-2-methylheptanoic acid (**12**) in ethanol microdroplets. b) Mass spectrum recorded in negative ion mode showing formation of product ions 2-methyl-6-oxoheptanoic acid (**[11 − H]**^−^, *m/z* 157) and 6-hydroxy-2-methylheptanoic acid (**[12 − H]**^−^, *m/z* 159). c) MS/MS of *m/z* 143 and the suggested structures of the fragment ions. d) MS/MS of *m/z* 157 and the suggested structures of its fragment ions.

**Figure S17.** a) Oxidation of cycloheptanone (**13**) to pimelic acid (**14**) in ethanol microdroplets. b) Mass spectrum recorded in negative ion mode showing formation of product ions pimelic acid (**[14 − H]**^−^, *m/z* 159) c) MS/MS of *m/z* 159 and the suggested structures of its fragment ions.

**Figure S18.** a) Oxidation of 1-indanone (**15**) to 2-(carboxymethyl) benzoic acid (**16**) in ethanol microdroplets. b) Mass spectrum recorded in negative ion mode showing formation of 2-(carboxymethyl) benzoic acid (**[16 − H]**^−^, *m/z* 179). c) MS/MS of *m/z* 165 and the suggested structures of the fragment ions. d) MS/MS of *m/z* 179 and the suggested structures of its fragment ions.

**Figure S19.** a) Oxidation of 2,2,6-trimethylcyclohexanone (**17**) to 2,2-dimethyl-6-oxoheptanoic acid (**18**) and 6-hydroxy-2,6-dimethylheptanoic acid (**19**) in ethanol microdroplets. b) Mass spectrum recorded in negative ion mode showing formation of 2,2-dimethyl-6-oxoheptanoic acid (**[18 − H]**^−^, *m/z* 171) and 6-hydroxy-2,6-dimethylheptanoic acid (**[19 − H]**^−^, *m/z* 173). c) MS/MS of *m/z* 173 and the suggested structures of the fragment ions. d) MS/MS of *m/z* 171 and the suggested structures of its fragment ions.

**Figure S20.** a) Oxidation of α-tetralone (**20**) to 2-(2-carboxyethyl) benzoic acid (**21**) in ethanol microdroplets. b) Mass spectrum recorded in negative ion mode showing formation of 2-(2-carboxyethyl) benzoic acid (**[21 − H]**^−^, *m/z* 193). c) MS/MS of *m/z* 179 and the suggested structures of the fragment ions. d) MS/MS of *m/z* 193 and the suggested structures of its fragment ions.

**Figure S21.** ^1^H NMR spectra of collected product in DMSO-d_6_ recorded using a 300MHz NMR instrument.

**Figure S22.** ^1^H NMR spectra of standard adipic-acid in DMSO-d_6_ recorded using a 300MHz NMR instrument.

**Figure S23.** ^1^H NMR spectra of cyclohexanone in DMSO-d_6_ recorded using a 300MHz NMR instrument.

**Figure S24.** ^1^H NMR of cyclohexanone (in DMSO-d_6_) after stirring overnight in ethanol under oxygen atmosphere; recorded using 300 MHz instrument.

**Figure S25.** (a) ESSI mass spectrum of cyclohexanone in negative ion mode (using oxygen as nebulizing gas, flow rate: 15 μL/min, gas pressure: 80 psi) without application of any voltage, showing adipic acid anion formation. (b) MS/MS data for *m/z* 145 confirms the structure of the adipic anion.
